# Introgression of a Complex Genomic Structural Variation Causes Hybrid Male Sterility in *GJ* Rice (*Oryza sativa* L.) Subspecies

**DOI:** 10.3390/ijms232112804

**Published:** 2022-10-24

**Authors:** Na Xu, Hai Xu, Zhengjin Xu, Fengcheng Li, Quan Xu

**Affiliations:** Rice Research Institute, Shenyang Agricultural University, Shenyang 110866, China

**Keywords:** *Oryza* *sativa* L., hybrid sterility, copy number variation, DUF1618 protein, genome introgression

## Abstract

Hybrids between different subspecies of rice *Oryza* *sativa* L. commonly show hybrid sterility. Here we show that a widely planted commercial *japonica*/*GJ* variety, DHX2, exhibited hybrid sterility when crossing with other *GJ* varieties. Using the high-quality genome assembly, we identified three copies of the *Sc* gene in DHX2, whereas Nipponbare (Nip) had only one copy of *Sc*. Knocking out the extra copies of *Sc* in DHX2 significantly improved the pollen fertility of the F_1_ plant of DHX2/Nip cross. The population structure analysis revealed that a slight introgression from Basmati1 might occur in the genome of DHX2. We demonstrated that both DHX2 and Basmati1 harbored three copies of *Sc*. Moreover, the introgression of *GS3* and *BADH2*/*fgr* from Basmati1 confers the slender and fragrance grain of DHX2. These results add to our understanding of the hybrid sterility of inter-subspecies and intra-subspecies and may provide a novel strategy for hybrid breeding.

## 1. Introduction

For over 2000 years, two major types of *O. sativa*—*O. sativa indica*/*Xian* (*XI*) and *O. sativa japonica*/*Geng* (*GJ*) Group—have historically been recognized [1]. There is great breeding potential in *XI*/*GJ* hybridization. Its hybrid F_1_ has strong heterosis in yield, quality, and stress resistance [2]. However, due to the semi-sterile characteristics of the *XI*/*GJ* hybrid, *XI*/*GJ* heterosis has encountered great difficulties in practical application, including a series of problems, such as high plant height, difference in flowering time, and low seed setting rate. The most important one is the low seed setting rate (pollen fertility) of the F_1_ generation of the *XI*/*GJ* hybrid [3]. Kato et al. (1928) showed that the average fertility of hybrids among cultivated rice varieties was more than 50%, while the fertility of *XI*/*GJ* F_1_ Hybrid was between 0% and 33% [4]. A series of subsequent studies showed that different *XI*/*GJ* hybrid combinations contained different sterile loci. The fertility of F_1_ generation of *XI*/*GJ* inter subspecies hybrid varied greatly from complete sterility to complete fertility, and the seed setting rate of the inter-subspecies hybrid was significantly lower than that of the intra-subspecies hybrid [5].

As early as 1962, Yang et al. proposed that *XI*/*GJ* hybrid rice can be used as a common strategy for rice breeding, and further explored the breeding methods regarding the improvement of seed setting rate of *XI*/*GJ* offspring, combination of the advantages of *XI* and *GJ* rice and heterosis utilization [6]. In 1987, Yuan et al. divided hybrid rice breeding into three development stages from the level of heterosis: intra-subspecies, inter-subspecies and utilization of distant heterosis, and strategically put forward the idea of hybrid rice breeding [7]. Because of the close relationship of intra-subspecies, the heterosis is limited in yield. The yield potential of an *XI*/*GJ* F_1_ hybrid is expected to be an effective way to further improve the yield of hybrid rice. The main limitation of direct utilization of strong *XI*/*GJ* heterosis is hybrid sterility [8]. Tremendous efforts have been made to overcome inter-subspecific hybrid sterility in rice, and genetic studies have identified approximately 50 loci involved in hybrid sterility [9]. The *XI*/*GJ* hybrid sterility is mainly affected by the genes at *Sc*, *S5*, *SA*, *hsa1*, *S7*, *dpl1*/*dpl2*, and *S27*/*S28* loci [10,11,12,13,14,15]. The isolation and cloning of these hybrid sterile genes have deepened the understanding of the molecular mechanism of plant reproductive isolation. The discovery of its genetic and molecular mechanism provides a new opportunity for overcoming hybrid sterility and utilizing heterosis.

**Figure 1:**
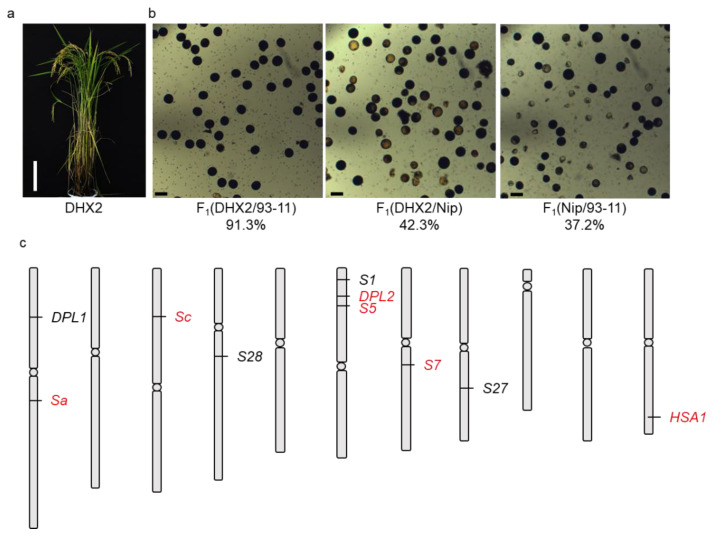
DHX2 exhibited hybrid sterility when crossing with other *GJ* varieties (**a**) The plant architecture of *GJ* variety DHX2. Scale bar: 20 cm; (**b**) The pollen phenotypes of F_1_ plants derived from the cross of DHX2/93-11, DHX2/Nip, and Nip/93-11. Scale bar: 50 μm; (**c**) The loci for hybrid sterility. The loci that have been cloned from *XI*/*GJ* cross were highlighted in red color.

Daohuaxiang2 (DHX2) is a large-scale variety planted in northern China. DHX2 is favored by the market because of its slender and fragrant grain. Thus, DHX2 was used as a backbone parent to breed new varieties in northern China (Figure 1a). During the breeding process, partial sterility due to the abortion of pollen was observed in the cross between DHX2 and other *GJ* varieties, whereas the F_1_ pollen derived from the cross between DHX2 and *XI* varieties showed normal fertility. In this study, we demonstrated that the semi-sterility between DHX2 and *GJ* was caused by introgression of a copy number variation at the *Sc* locus from Basmati1. The F_1_ hybrid sterility was rescued when knocking out an extra copy of *Sc* in DHX2 using CRSIPR gene editing technology. Our results identified a novel allele of *Sc* and will provide an effective approach to conduct crossbreeding using DHX2 as a parent line.

## 2. Results

### 2.1. F_1_ Hybrid Sterility between Geng Varieties

**Figure 2:**
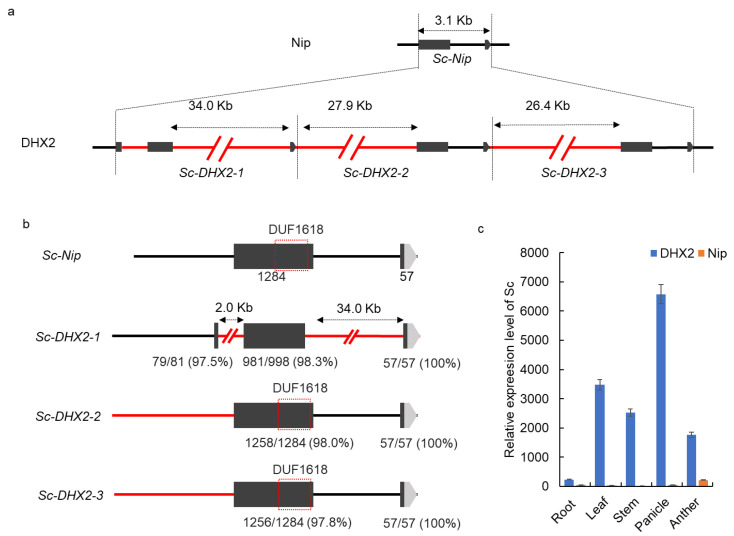
The structure variation of DHX2 at the *Sc* locus. (**a**) The copy number variation of the *Sc* locus in DHX2 and Nip; (**b**)the sequence variation of three copies of *Sc* in DHX2. The red line indicates the sequence of DHX2 is different from Nip; (**c**) the expression level of *Sc* on various tissues of DHX2 and Nip.

Rice hybrid sterility occurs extensively in the hybrid between *XI* and *GJ* [16]. For instance, the pollen fertility of the F_1_ hybrid between *GJ* variety Nipponbare (Nip) and *XI* variety 93–11 was 37.2% (Figure 1b). DHX2 is a *GJ* variety with slender and fragrant grain and was widely planted in northern China. Interestingly, F_1_ hybrid sterility was observed in the cross between DHX2 and other *GJ* varieties, such as Nip (Figure 1b). Moreover, the pollen fertility of the F_1_ hybrid derived from the cross between DHX2 and an *XI* variety 93–11 was significantly improved compared to the cross between Nip and 93–11 (Figure 1b). As a recent study has published the de novo genome assembly of DHX2 [17], we compared the sequence of published hybrid causal genes in an *XI*/*GJ* hybrid (Figure 1c). The result showed that the sequences of *Sa*, *DPL2*, *S5*, *S7*, and *HSA1* in DHX2 were identical to those of Nip. However, we found a complex structural variation at the *Sc* locus in DHX2 compared to Nip (Figure 2a).

### 2.2. Complex Genomic Structural Variation in the Sc-DHX2 Alleles

**Figure 3:**
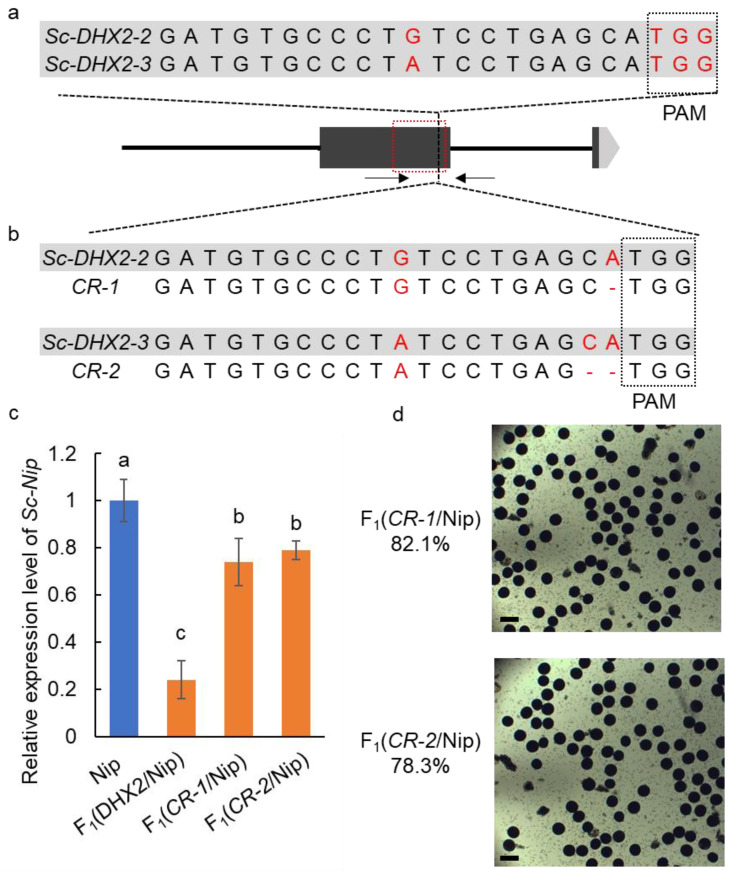
The CRISPR/Cas9 gene editing of the extra copy of *Sc* in DHX2. (**a**) The sgRNA sequence of *Sc-DHX2-2* and *Sc-DHX2-3*. The red dash line box indicates the DUF1618 domain; (**b**) the sequence of two mutant lines; (**c**) the expression level of *Sc-Nip* in the anthers of the F_1_ plants. Different letters denote significant differences (*p* < 0.05) from Duncan’s multiple range tests; (**d**) the pollen phenotypes of F_1_ plants derived from the cross of *CR-1*/Nip and *CR-2*/Nip. Scale bar: 50 μm.

The copy number variation at the *Sc* locus was reported to confer the *XI*/*GJ* hybrid male sterility [15]. Normally, the *GJ* allele (*Sc-Nip*) contains a pollen-essential gene encoding a DUF1618 domain protein, whereas the *XI* allele harbors at least two extra copies besides *Sc-Nip*. Here, we found that there were three tandem duplicated segments, and each of them contained a copy of *Sc* homolog protein (*Sc-DHX2-1*, *Sc-DHX2-2*

, and *Sc-DHX2-3*) in DHX2 (Figure 3a). The *Sc-DHX2-1* shared a similar promoter sequence to *Sc-Nip*, but lost the DUF1618 domain due to the 2.0 kb insertion in the first exon and a 34.0 kb insertion at the intron of *Sc-DHX2-1*. Thus, we concluded that the *Sc-DHX2-1* is a pseudogene. The other two copies, *Sc-DHX2-2* and *Sc-DHX2-3*, each contain the entire sequence of *Sc-Nip*. However, there are several SNPs in the exon of *Sc-DHX2-2* and *Sc-DHX2-3* compared to that of *Sc-Nip*. The first exon of *Sc-Nip* contains 1284 bp, the first exon of *Sc-DHX2-2* was 98.0% (1258/1284), identical to *Sc-Nip*, and the first exon of *Sc-DHX2-3* was 97.8% (1256/1284), identical to *Sc-Nip* (Figure 3b). We then checked whether the extra copies of *Sc* were altering the expression pattern and expression level of *Sc*. The RT-PCR showed that *Sc* was specifically expressed in anther with a low level in Nip, whereas, they were broadly in leaf, stem, panicle, and anther, with a significantly higher level in DHX2 compared to Nip (Figure 3c). Thus, we hypothesized that the copy number variation at the *Sc* locus in DHX2 conferred the F_1_ hybrid sterility when DHX2 was crossed to other typical *GJ* varieties.

### 2.3. Knockout of Sc-DHX2-2 or Sc-DHX2-3 Rescues the F_1_ Hybrid Sterility

To confirm whether the extra copies of *Sc-DHX2-2* and *Sc-DHX2-3* caused the F_1_ hybrid sterility of the cross between DHX2 and other *GJ* varieties, we used the CRISPR/cas9 plant genome editing system to knockout the *Sc-DHX2-2* and *Sc-DHX2-3* in DHX2. As there are two SNPs between *Sc-DHX2-2* and *Sc-DHX2-3*, we designed the different sgRNA for *Sc-DHX2-2* and *Sc-DHX-3* based on the SNPs (Figure 3a). By transforming DHX2 with these two constructs, we successfully identified a plant CR-1 with 1bp deletion at the first exon of *Sc-DHX2-2*, and a plant CR-2 with 2 bp at the at first exon of *Sc-DHX2-3*. The homozygous mutants of *Sc-DHX2-2* and *Sc-DHX2-3* did not exhibit a significant difference of agronomic traits compared to DHX2. We then crossed CR-1 and CR-2 with Nip to generate F_1_ plants (Figure 3b). The *Sc-Nip* expression level of F_1_ (CR-1/Nip) and F_1_ (CR-2/Nip) was significantly higher than that of F_1_ (DHX2/Nip) (Figure 3c), and the pollen fertility of these F_1_ plants was significantly improved (Figure 3d).

### 2.4. Introgression from Basmati Variety

**Figure 4:**
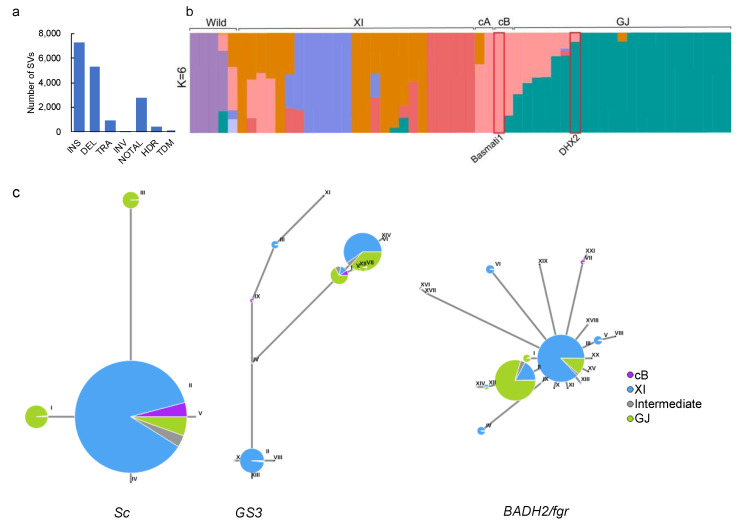
The population structure analysis of 58 assemblies. (**a**) The different types of SVs between DHX2 and Nip. INS: insertion, DEL: deletion, TRA: translocation, INV: inversion, NOTAL: not aligned region, TDM: tandem repeat, HDR: highly diverged regions; (**b**) Structure analysis of 58 accessions with different numbers of clusters, K = 6. The different colors represent different groups of rice accessions. (**c**) Haplotype network of *Sc*, *GS3*, and *BADH2*/*fgr* using sequence data of the 3000 Rice Genomes Project. Circle size is proportional to the sample number for a given haplotype. Different colors represent ecotypes, as shown in the illustration. Different Roman numerals indicate various haplotypes of *Sc*, *GS3*, and *BADH2*/*fgr*.

**Figure 5:**
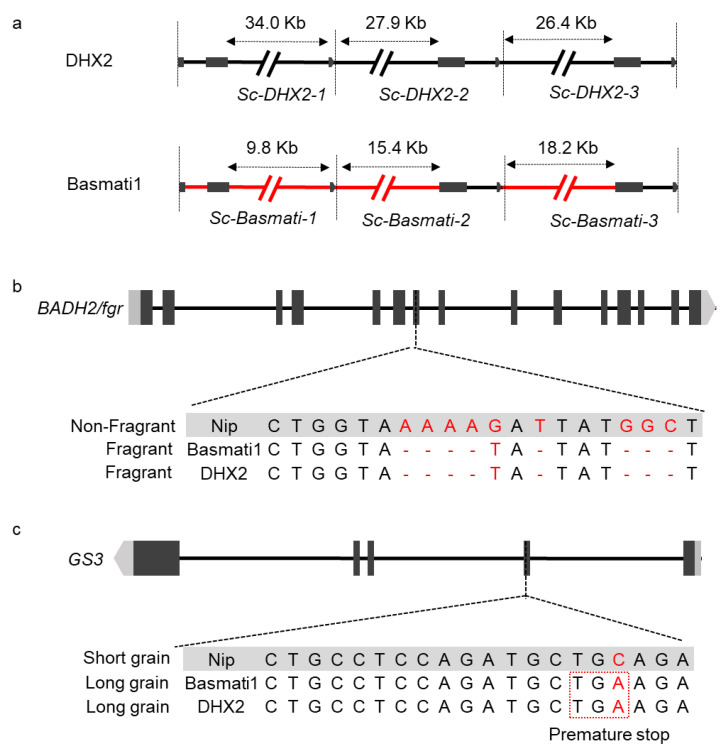
The introgression loci in DHX2 from Basmati1. (**a**) The structural variation of the *Sc* locus in DHX2 and Basmati1. The red line indicates that the sequence of Basmati1 is different from DHX2; (**b**) the sequence variation of *BADH2*/*fgr* in DHX2, Nip, and Basmati1; (**c**) the sequence variation of the *GS3* locus in DHX2, Nip, and Basmati1.

As most GJ varieties only have a single copy of *Sc* [15], we hypothesized that the copy number variation of the *Sc* locus in DHX2 was caused by introgression from *XI* or other subspecies. We first compared the genome of DHX2 to Nip and identified 16,873 SVs (Figure 4a). Then we collected 58 long-read *de novo* assemblies to conduct a population structure analysis [17,18,19]. The result showed that the slight introgression from the circum-Basmati group (cB) might occur in the genome of DHX2 (Figure 4b). The cB group comprises the famous Basmati and Sadri aromatic varieties which we term the circum-Basmati group (cB) [1]. Basmati1 is a high-yield Basmati variety, that possesses extra-long slender grains, a pleasant aroma, appealing taste, good mouthfeel, and easy digestibility [20]. Considering that DHX2 exhibited similar characteristics to Basmati1, such as slender and fragrance grain, we speculated that DHX2 inherited these characteristics from Basmati1. As the truncated *GS3* protein contributed to the slender grain of Basmati1 [21], and the fragrance of Basmati1 was regulated by gene *BADH2*/*fgr* [22,23], we conducted the haplotype network analysis of *Sc*, *GS3*, and *BADH2*/*fgr* using sequence data of the 3000 Rice Genomes Project [24]. The results indicated that *Sc*, *GS3*, and *BADH2*/*fgr* differentiated among cB, *XI*, and *GJ* groups (Figure 4c). We subsequently compared the sequence of the *Sc* locus between DHX2 and Basmati1. The result showed that both DHX2 and Basmati1 had three copies of *Sc*, although the interval of the three copies was different between DHX2 and Basmati1 (Figure 5a). The *BADH2*/*fgr* of DHX2 was identical to that of Basmati1, which was different to other *GJ* variety, such as Nip (Figure 5b). We subsequently compared the sequencing of *GS3* between DHX2 and Basmati1. The result exhibited that both DHX2 and Basmati1 shared a C/A SNP compared to Nip. The C/A SNP generated a premature stop codon, which caused a frameshift mutation in the C terminus that yielded a truncated protein of GS3 (Figure 5c).

## 3. Discussion

A combination of the advantages of *XI* and *GJ* rice through the *XI*/*GJ* crossbreeding was the basic breeding strategy in northern China. Hybridization between *XI* and *GJ* rice combined with the utilization of the ideal plant type has led to the development of high-yielding *GJ* rice in northern China. Crossbreeding causes genome introgression from *XI* and cB varieties. Our previous study revealed that the *XI* pedigree introgression frequencies were significantly increased in cultivars bred after 1990 and the *XI* pedigree introgression frequencies were significantly positively correlated with grain number per panicle [25]. In an investigation of 1200 Chinese accessions, an average of 6.8 Mb *XI* genome introgression was found in *GJ* accessions [26]. Notably, the important gene haplotypes controlling plant architecture, yield components, and pest and disease resistance, including *IPA1*, *SMG1*, *DEP3*, *Pib*, *Pi-d*2, and *Bph3*, were introduced from *XI* rice to *GJ* by introgression [27]. In this study, we found that there is cB introgression in some Chinese GJ varieties, such as DHX2. The introgression of *Sc*, *GS3,* and *BADH2/fgr* from cB variety Basmati1 caused the special characteristics of DHX2 in *GJ*/*GJ* hybrid sterility, slender grain shape, and fragrance.

Copy number variations are widely distributed in plant genomes [28]. Recently reported pan-genomes have revealed hidden copy number variations and demonstrated that copy number variations regulate important agronomic traits [17,29,30]. The copy number variation at the *GL7* locus contributes to the grain size diversity in rice [31], the extra copy number at the *GNP1* locus significantly increased the grain number per panicle [30], the dual copies of *OsMADS18* are likely a causal candidate accounting for the early flowering phenotype Koshihikari [17]. However, only the copy number variation of *Sc* differentiated between *XI* and *GJ* subspecies [15]. Our study detected a *GJ* variety DHX2 harboring three tandem copies at the *Sc* locus, which might be caused by the introgression of Basmati1. Moreover, the sequence of the extra copies of *Sc* showed a slight difference compared to the *XI* allele [15]. These findings suggested that the presence of rich copy number variation is a treasure of the total genetic diversity of *O.sativa*, revealing that the copy number variation originated from the intricate breeding history.

DUF1618 is a new gene family that originated after the dicot–monocot divergence. DUF1618 family members in plants possess a 56–199-amino acid conserved domain, and there are 121 DUF1618 genes identified in the rice genome [32]. Recent studies have demonstrated that the DUF1618 gene family is involved in the regulation of hybrid sterility. The *GJ* type allele of *HSA1a* encodes a highly conserved plant-specific domain of DUF1618, whereas the *XI* type allele has two deletion mutations that cause disruption of domain structure. The recombinant haplotype of *HSA1a* and *HSA1b* caused semi-sterility [14]. Shen et al. (2017) genotyped 14 *GJ* cultivars and 21 *XI* cultivars and found that all tested *GJ* type alleles of *Sc* contain a pollen-essential gene encoding a DUF1618 domain, and all tested *XI* type alleles contain two or three tandem duplicated *Sc* segments. The genomic structural variation at the *Sc* locus causes hybrid male sterility between *XI* and *GJ* [15]. Our study found that there are copy number variations at the *Sc* locus in *GJ* variety DHX2 and the extra two copies of *Sc* cause hybrid male sterility when DHX2 is crossed to other *GJ* varieties. However, only these two genes have been functionally studied among 121 DUF1618 gene families. The function of the other 119 DUF1618 genes needs to be further studied.

## 4. Materials and Methods

### 4.1. Plant Materials

In this study, the *GJ* variety DHX2, *GJ* variety Nipponbare (Nip), and *XI* variety 93-11, and the F_1_ plants were employed. Shenyang Agricultural University’s Rice Research Institute (41° N, 123° E) was used to conduct field tests. The seeds were sowed on April 16 and transplanted to the field on May 22 in 2020. Each line was planted in three rows, with 10 plants per row and a 30 cm × 13.3 cm plant spacing.

### 4.2. Pollen Fertility Test

Five young panicles of each F_1_ plant were sampled. Pollen fertility was evaluated using pollen grains stained with I_2_KI solution. We counted 500 pollen to distinguish the stained/unstained pollen grains for each young panicle under a microscope. The average data of five young panicles was presented in the Figure 1 and Figure 3.

### 4.3. Expression Analysis

The pollen was sampled according to the method described previously [15]. The primer for the RT-PCR of *Sc* and *Actin* was listed in Table S1. The investigation was conducted by three biological replicates, and the significance was analyzed by Duncan’s multiple range tests.

### 4.4. Vector Construction and Plant Transformation

To conduct the CRISPR/Cas9 gene editing, the vector construction was performed as described by Li et al. [33]. We designed the specific single-guide RNA (sgRNA) sequences targeting the *Sc-DHX2-2* and *Sc-DHX2-3* copies. The specificity of the targeting sequence was confirmed by BLAST searching against the Nip genome [34]. The rice transformation was conducted as described elsewhere [35]. We extracted the genomic DNA from transformants, and the genomic DNA was sequenced for mutant identification. The PCR products (200–500 bp) were sequenced and identified using the degenerate sequence decoding method [36]. We examined 20 independent transgenic plants for each construction at T_0_ generation. The sequence analysis detected 12 and 9 plants with mutations of *Sc-DHX2-2* and *Sc-DHX2-3*, respectively. Among these mutants, 3 and 4 plants were putative homozygous mutants, of *Sc-DHX2-2* and *Sc-DHX2-3*, respectively.

### 4.5. Population Structure Analysis

We collected 55 de novo assembled genomes of wild type, *XI*, *GJ*, circum-Aus group, (cA) which encompasses the Aus, Boro, and Rayada ecotypes from Bangladesh and India, and cB, based on the long-read sequencing techniques [17,18,19]. After discarding duplicated assemblies, a total of 57 assembled genomes were compared to the genome of Os-Nipponbare-Reference-IRGSP-1.0 (https://rapdb.dna.affrc.go.jp/download/irgsp1.html) (Kawahara et al., 2013) using MUMmer (v 4.0) [37]. After filtering the low-quality structure variations using metrics of minor allele frequency (MAF) > 0.05′ from the raw SV dataset, we retained 156,319 high-confidence SVs for population structure analysis. Detailed information on SVs was described in our previous study [30]. The population genetic structure was examined using the program ADMIXTURE (v1.23) [38] with K values (the putative number of populations) from 2 to 10. The K = 6 values were chosen to display the genetic admixtures of rice populations. A haplotype network of *Sc*, *GS3*, and *BADH2*/*fgr* was conducted using the tools of Haplotype Network Analysis on RiceVarMap v2.0 (http://ricevarmap.ncpgr.cn/hap_net/ (accessed on 22 September 2022)) [39].

## Data Availability

The study did not report any data.

## Supplementary Materials

The supporting information can be downloaded at: https://www.mdpi.com/article/10.3390/ijms232112804/s1.
